# Physical activity, body functions and disability among middle-aged and older Spanish adults

**DOI:** 10.1186/s12877-017-0551-z

**Published:** 2017-07-18

**Authors:** Alexandre Caron, Alba Ayala, Javier Damián, Carmen Rodriguez-Blazquez, Javier Almazán, Juan Manuel Castellote, Madgalena Comin, Maria João Forjaz, Jesús de Pedro, on behalf of the DISCAP-ARAGON research group

**Affiliations:** 1 University of Lille, EA 2694 - Department of Public Health, F-59000 Lille, France; 2 Epidemiology and Biostatistics Department, National School of Public Health, and Health Service Research Network for Chronic Diseases (Red de Investigación en Servicios de Salud en Enfermedades Crónicas/REDISSEC), Carlos III Institute of Health (Instituto de Salud Carlos III), Avda Monforte de Lemos 5, 28029 Madrid, Spain; 3 National Center of Epidemiology and Consortium for Biomedical Research in Neurodegenerative Diseases (Centro de Investigación Biomédica en Red sobre Enfermedades Neurodegenerativas/CIBERNED), Carlos III Institute of Health, Madrid, Spain; 4 National School of Occupational Medicine and Consortium for Biomedical Research in Neurodegenerative Diseases/CIBERNED, Carlos III Institute of Health Madrid Spain, Madrid, Spain; 5 School of Health Sciences, Zaragoza University, Zaragoza, Spain

**Keywords:** Physical activity, Disability evaluation, International classification of functioning, disability and health, Middle-aged and older adults

## Abstract

**Background:**

Physical activity (PA) is a health determinant among middle-aged and older adults. In contrast, poor health is expected to have a negative impact on PA. This study sought to assess to what extent specific International Classification of Functioning, Disability and Health (ICF) health components were associated with PA among older adults.

**Methods:**

We used a sample of 864 persons aged ≥50 years, positively screened for disability or cognition in a cross-sectional community survey in Spain. Weekly energy expenditure during PA was measured with the Yale Physical Activity Survey (YPAS) scale. The associations between body function impairment, health conditions or World Health Organization Disability Assessment Schedule (WHODAS 2.0) disability scores and energy expenditure were quantified using negative-binomial regression, and expressed in terms of adjusted mean ratios (*aMRs)*.

**Results:**

Mean energy expenditure was 4542 Kcal/week. A lower weekly energy expenditure was associated with: severe/extreme impairment of mental functions, *aMR* 0.38, 95% confidence interval, CI (0.21–0.68), and neuromusculoskeletal and movement functions, *aMR* 0.50 (0.35–0.72); WHODAS 2.0 disability, *aMR* 0.55 (0.34–0.91); dementia, *aMR* 0.45 (0.31–0.66); and heart failure, *aMR* 0.54 (0.34–0.87). In contrast, people with arthritis/osteoarthritis had a higher energy expenditure, *aMR* 1.27 (1.07–1.51).

**Conclusion:**

Our results suggest that there is a strong relationship between selected body function impairments, mainly mental, and PA. Although more research is needed to fully understand causal relationships, strategies to improve PA among the elderly may require targeting mental, neuromusculoskeletal and movement functions, disability determinants (including barriers), and specific approaches for persons with dementia or heart failure.

**Electronic supplementary material:**

The online version of this article (doi:10.1186/s12877-017-0551-z) contains supplementary material, which is available to authorized users.

## Background

The proportion of middle-aged and older adults in the populations of industrialized countries is expected to double within the next 30 years [1]. This group is characterized by a higher prevalence of chronic health conditions and disability [2], and a lower degree of physical activity (PA) [3]. PA is defined by the American Heart Association as, “any bodily movement produced by skeletal muscles that results in energy expenditure beyond resting expenditure” [4]. The literature is consistent with a significant reduction in morbidity through physical fitness and activity [5]. Energy expenditure associated with free-living activity is associated with a lower risk of mortality in healthy older adults [6]. It also reduces premature mortality, regardless of genetic and other familial factors [7]. Short-term effects on retaining fitness have been experimentally demonstrated [8].

Disability determinants can be described either from a diagnostic point of view with the *International Classification of Diseases*, or from a functional point of view using the *International Classification of Functioning, Disability and Health* (ICF) [9]. Both World Health Organization (WHO) classifications are complementary but the ICF has the advantage of not being diagnosis-centered. Nowadays, the WHO Disability Assessment Schedule (WHODAS 2.0) and the ICF-Checklist are important generic ICF-based tools for assessing disability [10, 11].

**Fig. 1:**
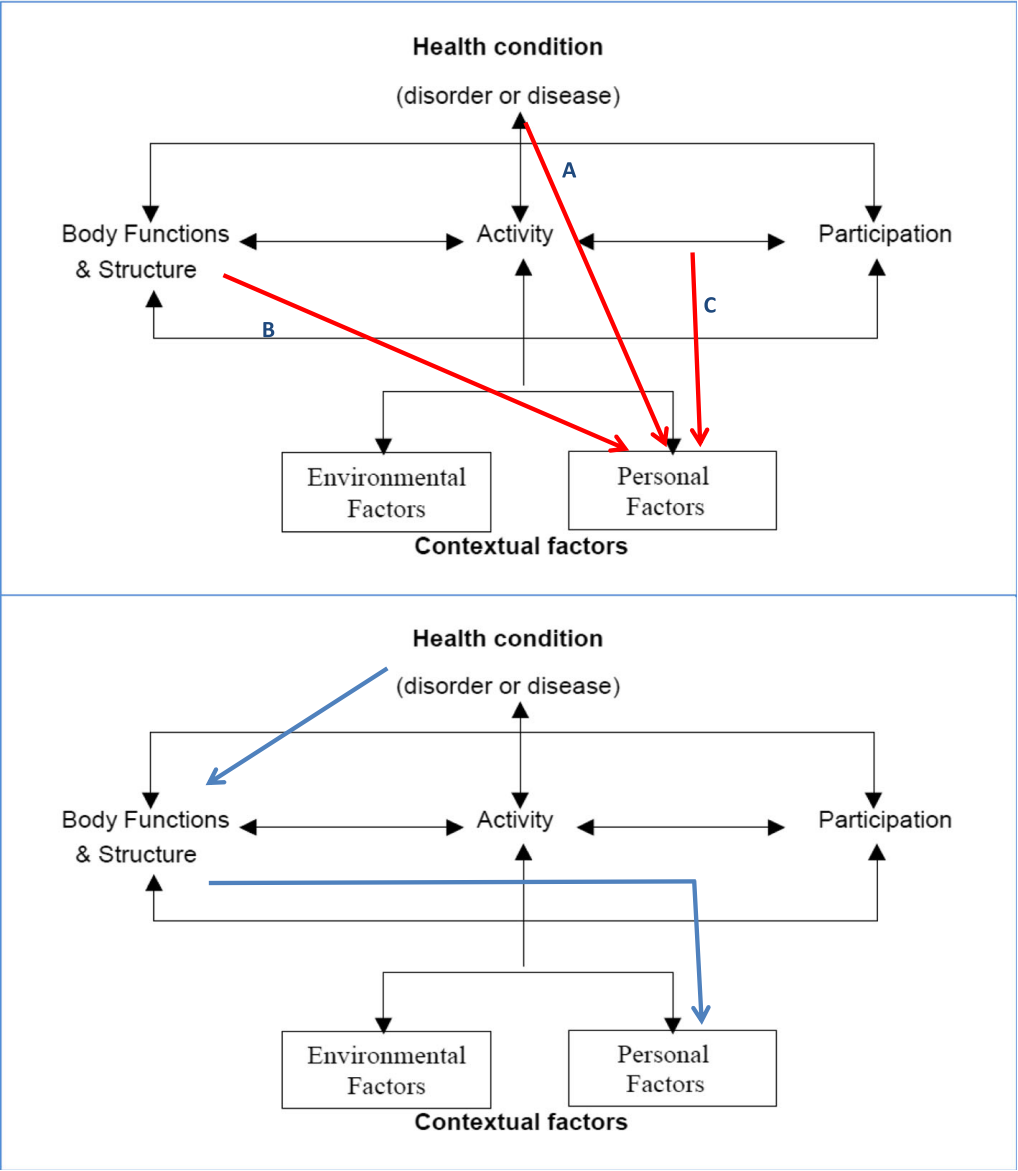
*Upper*. International Classification of Functioning, Disability and Health framework (ICF, adapted from the World Health Organization diagram): *arrows* indicate different statistical modeling approaches, taking physical activity as a personal factor potentially determined by health conditions (diagnoses), body functions, or activities and participation. The direction of *red arrows* indicates the role of independent, potentially causal variables and of physical activity as the dependent variable in models. *Blue arrows* suggest the main determinant and complex interplay of non-environmental determinants of physical activity in the ICF framework. *Lower*. Suggested main causal interpretation of study results from models, taking into account biologically plausible function loss and reported effect of function impairment on activity and participation. *Blue arrow* represents suggested temporal phenomena as intermediary steps in the causal chain

Relationships between PA and disability are complex. Higher PA has been shown to delay the onset of disability among healthy older adults [12]. However, a two-way relationship is plausible, i.e., once older adults start suffering disability, they are also likely to reduce PA. Difficulties in performing some activities (e.g., mobility, domestic tasks or work), when measured in time or energy expenditure, might be integrated into the ICF framework as a contextual personal factor (Fig. 1) [13]. Additionally, the absence of such difficulties is compatible with sedentary life or high energy expenditure in PA. Hence, PA was considered a personal factor, and its main associations with health conditions, body functions and structure, and activity and participation were modeled. Analyzing PA determinants under the ICF scope could highlight areas for improvement in the management of disability and promotion of PA. Accordingly, the aim of this study was to assess how specific ICF components, such as diagnoses, body function impairments and disability (activity and participation), might be related to the PA of a community sample of middle-aged and older people positively screened for disability and cognition.

## Methods

### Study design

We used a cross-sectional population-based survey conducted from 2008 through 2011 on older adults living in Cinco Villas County and Zaragoza city, both in the Aragon Region of North-East Spain. A complete description of the design, methods and population characteristics is given elsewhere [14]. The initial Cinco Villas County sample was expanded to include a sample from two health districts in the city of Zaragoza, in order to include rural, urban and institutionalized populations. A summary description of the rural and urban study sample is provided elsewhere [15].

### Participants

A random sample of 1707 individuals aged 50 years and over, 1202 from Cinco Villas and 505 from Zaragoza, was drawn from the Social Security card-holder register (which includes persons entitled to care under the public health system). The selected individuals were double screened for disability and cognitive function by trained research assistants, and those with comprehensive assessments were included in the analysis [15]. Screening involved two stages: firstly, we selected individuals with at least one positive answer in a disability questionnaire, the WHODAS 2.0 12-item [14]. Those with cognitive impairment, defined as a score < 24 points in the Spanish version of the Mini-Mental Status Examination (*Mini Examen Cognoscitivo [MEC]*) [16], were also deemed to have tested positive to screening. Secondly, those who had screened positive underwent an in-depth assessment of their PA, disability, depressive symptoms, medical history, and tobacco and alcohol consumption. In addition, a short physical examination was performed, and anthropometric measures (weight and height) were taken. Informed consent was obtained from all participants, or if this was not possible due to cognitive impairment, from close family relatives. Ethical aspects were approved by the Human Subjects Committee of the Aragon Health Sciences Institute.

### Measures

#### Physical activity

The primary outcome was energy expenditure as measured by the YPAS [17]. This self-reported questionnaire has been validated to measure energy expenditure among older adults in Spain, and has an adequate test-retest reliability and a satisfactory concurrent validity with Caltrac activity units [18]. It assesses weekly PA energy expenditure in kilocalories (kcal), and the total time index (hours/week) based on the latest week’s PA [19]. The YPAS includes questions on the following physical-activity categories: work and activities (shopping, climbing stairs with weight, laundry, food preparation, home repair, housework, etc.); yard work (gardening, lawn mowing, etc.); care taking (children, or older or disabled people); exercise (brisk walking, swimming, aerobics, etc.); and recreation (leisure walking, dancing, golf, etc.). Energy expenditure was computed by multiplying the time (in hours per week) spent doing each PA by the individual’s body weight and an intensity code [20], and then summing this to obtain the energy expenditure index in kcal/week [21]. Cases with an energy expenditure higher than 32,000 kcal/week (over 4500 kcal/day), or more than 120 h/week (17 h a day) of PA were deemed to be outliers and therefore discarded.

#### Disability

##### Activity and participation

Disability domains were assessed using the WHODAS 2.0 36-item, 2010 version [22]. This was developed to assess disability via questions designed to ascertain the degree of difficulty experienced by someone when performing activities in the following dimensions: understanding and communicating; getting around; self-care; getting along with people; life activities; and participation in society. Each of the 36 items is coded with a 5-level scale, ranging from none to mild, moderate, severe and extreme difficulty [22]. Higher scores indicate more severe disability.

##### Body function

This was measured with the ICF Checklist [11], a semi-structured guide designed to help trained personnel assess and record data on major ICF categories (body functions and structures, activities and participation, as well as contextual environmental factors, though the latter were not addressed here), after examining diagnoses present in primary-care medical records. For study purposes, we used only first-level category codes (b1 to b8, see Additional file 1), encompassing (b1) mental functions, (b2) sensory functions and pain, (b3) voice and speech, (b4) functions of the cardiovascular, hematological, immunological and respiratory systems, (b5) functions of the digestive, metabolic and endocrine systems, (b6) genitourinary and reproductive functions, (b7) neuromusculoskeletal- and movement- related functions, and (b8) functions of the skin and related structures. Impairment was initially measured using global scores for each function (e.g., b1-mental), obtained by averaging component scores (e.g., b110 to b167, nine items) and assigned a discrete value by rounding (standard rules) the resulting mean [15]. ICF ordinal scores were generated, ranging from 0-no impairment, to 1-mild, 2-moderate, 3-severe and 4-complete impairment. The degree of impairment was further grouped as: no/mild; moderate; or severe/complete.

#### Confounding variables

Socio-demographic variables included sex and age. We also gathered data on rural (Cinco Villas) or urban (Zaragoza city) setting, and whether the participant was institutionalized. Medical history was obtained from primary care records and then categorized by trained staff into 24 chronic health conditions [23]. We computed the number of chronic diseases. Body mass index (BMI) was computed as weight in kilograms divided by the square of height in meters (kg/m^2^). Cognitive status was assessed using the Mini-Mental Status Examination [16].

### Statistical analysis

The analytical strategy was based on the ICF framework (Fig. 1). The main associations were quantified using regression models (represented by red arrows in the top figure), as: (a) health conditions with PA; (b) body function impairment with PA; and (c) WHODAS 2.0 disability with PA. In view of the fact that, in a previous paper, we had reported strong WHODAS 2.0 associations with domains (b1) and (b7), i.e., mental function and neuromusculoskeletal and movement function impairment, respectively [15], we refrained from using WHODAS 2.0 as a confounder in models. As a first approach, we performed a descriptive analysis of the sample characteristics. We stratified the sample by sex, and compared the means (Student’s t-tests) and proportions (chi-square or Fisher’s exact tests). YPAS energy expenditure (in kcal/week) was used as the primary outcome variable [17, 24]. We used negative binomial regression models to estimate the associations between energy expenditure (in kcal/week, a rate of a count) and the main independent variables, i.e., health conditions, ICF body functions, and WHODAS 2.0 disability. These models allow for computation of the adjusted mean ratios (*aMRs*) between index and reference categories. For instance, in the case of energy expenditure, an *aMR* of 1.05 for women would mean that women expended 5% more energy than did men. Negative binomial models also enabled us to correct for overdispersion of energy expenditure distribution and to explore the dose-response relationship between the level of PA and the extent of body function impairment. Moreover, a linear model was rejected because residuals failed to meet statistical assumptions. We adjusted for the following potential confounders: sex and age (for which adjustment is important, since the YPAS uses an absolute measure of PA intensity); urban or rural setting; institutionalization status (yes/no); number of chronic diseases (excluding the index disease in any analysis involving that disease); and cognitive status (MEC score, though not when dementia was analyzed). We used total PA time (hours/week) as an outcome for a sensitivity analysis. These latter analyses included BMI as an adjusting variable.

## Results

### Sample characteristics

**Fig. 2:**
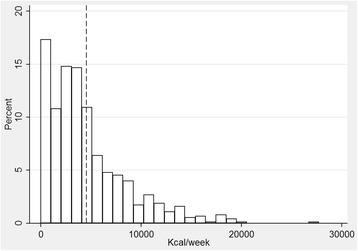
Distribution of positively screened sample by energy expenditure, as measured by the Yale Physical Activity Survey scale – *dashed line* shows the mean energy expenditure value

Of the 1707 participants, 864 screened positive and comprised the study sample. Participants had a mean (M) age (standard deviation, SD) of 73.2 (11.4) years. Women had more health conditions than did men (*p* = 0.004). The mean (SD) disability score was 28.4 (21.1), with higher mean scores among women than among men (*p* = 0.002). Energy expenditure, as measured by the YPAS, differed by sex, being 3851 kcal/week in men versus 4914 kcal/week in women (*p* = 0.001). Mean (SD) PA duration in hours/week was also lower in men, 13.9 (13.5) hours, than in women, 22.0 (17.6) hours, *p* < 0.001. Similarly, the pattern of PA in the three main categories (work, yard work, and recreation) was different between men and women: while women expended most PA calories performing work activities (3622 of a total of 4913 kcal, and 17 of a total of 22 h), none of these three categories predominated among men. The distribution of the positively screened sample by energy expenditure is shown in Fig. 2. The risk profile followed a zero-inflated Poisson function, with negligible Y-value differences between categories of energy expenditure below the mean value. Approximately one third engaged in very little PA. Table 1 shows the sample characteristics.

**Table 1:** Characteristics of the positively screened sample, by sex

	Total (864)	Men (*n* = 301)	Women (*n* = 563)	*p*-value
Age, years, *n* (%)				0.520
50–64	215 (24.9)	76 (25.4)	139 (24.7)	
65–79	360 (41.8)	131 (43.8)	229 (40.7)	
≥ 80	287 (33.3)	92 (30.8)	195 (34.6)	
Setting, *n* (%)				0.026
Rural	635 (73.5)	235 (78.1)	400 (71.1)	
Urban	229 (26.5)	66 (21.9)	163 (28.5)	
Institutionalization, *n* (%)				0.238
No	799 (92.5)	274 (91.0)	525 (93.3)	
Yes	65 (7.5)	27 (9.0)	38 (6.8)	
Body Mass Index (kg/m^2^), n (%)				0.262
≤ 24	170 (23. 5)	53 (20.8)	117 (24.7)	
25–30	306 (42.0)	117 (45.9)	189 (40.0)	
≥ 30	252 (34.6)	85 (33.3)	167 (35.3)	
Cognitive impairment (MEC < 24), *n* (%)				
No	684 (80.5)	241 (82.0)	443 (79.7)	
Yes	166 (19.5)	53 (18.0)	113 (20.3)	
WHODAS 2.0; *n* (%)				
No disability	30 (3.5)	13 (4.4)	17 (3.1)	0.039
Mild disability	441 (51.5)	164 (55.0)	277 (49.6)	
Moderate disability	247 (28.9)	87 (29.2)	160 (28.7)	
Severe disability	138 (16.2)	34 (11.4)	104 (18.6)	
YPAS total time index (hours/week) (*n* = 848), mean (SD)	19.2 (16.7)	13. 9 (13.5)	22.01 (17.6)	<0.001
Care taking	0.60 (3.18)	0.50 (3.03)	0.65 (3.26)	0.521
Exercise	0.60 (1.70)	0.66 (1.75)	0.57 (1.67)	0.486
Recreation	4.40 (5.26)	4.54 (5.26)	4.32 (5.26)	0.557
Work	12.43 (15.61)	4.53 (7.60)	16.63 (17.08)	<0.001
Yard work	1.52 (6.07)	3.65 (9.55)	0.38 (2.08)	<0.001
YPAS energy expenditure (kcal/week) (*n* = 750), mean (SD)	4542 (3991)	3854 (3924)	4913 (3981)	0.001
Care taking	208 (1074)	185 (1154)	220 (1029)	0.667
Exercise	209 (681)	256 (768)	184 (630)	0.166
Recreation	953 (1084)	1133 (1299)	857 (936)	<0.001
Work	2740 (3231)	1102 (1708)	3622 (3503)	<0.001
Yard work	515 (2161)	1295 (3448)	96 (547)	<0.001

*MEC* Mini-Examen Cognoscitivo (Spanish version of the Mini-Mental Status Examination (MMSE)), *YPAS* Yale Physical Activity Survey

### Sample distribution by level of impairment

When the sample distribution was analyzed by level of impairment, measured by the ICF-Checklist in eight body function domains (Table 2), proportions generally tended to decrease with increasing impairment. However, a high proportion of the sample (48.6%) presented with high functional impairment in genitourinary-and- reproductive functions. Noticeable differences between women and men were observed for voice-and-speech, digestive-and-endocrine functions, and neuromusculoskeletal-and-movement functions. Compared to men, women more frequently experienced severe/extreme voice-and-speech as well as moderate digestive-and-endocrine impairment and neuromusculoskeletal impairment.

**Table 2:** Sample distribution by ICF Checklist body function domains, degree of impairment, and sex

Body function degree of impairment	Total, *N* (%)	Men, *N* (%)	Women, *N* (%)	*P*-value*
Mental				
None or mild	533 (62.1)	190 (63.8)	343 (61.3)	0.268
Moderate	262 (30.5)	92 (30.9)	170 (30.4)	
Severe or extreme	63 (7.3)	16 (5.4)	47 (8.4)	
Voice and speech				
None or mild	95 (11.1)	44 (14.8)	51 (9.1)	<0.001
Moderate	597 (69.7)	215 (72.4)	382 (68.2)	
Severe or extreme	165 (19.3)	38 (12.8)	127 (22.7)	
Sensory and pain				
None or mild	770 (90.8)	263 (89.2)	507 (91.7)	0.360
Moderate	40 (4.7)	18 (6.1)	22 (4.0)	
Severe or extreme	38 (4.5)	14 (4.7)	24 (4.3)	
Cardiovascular and respiratory				
None or mild	545 (63.5)	183 (61.4)	362 (64.6)	0.536
Moderate	292 (34.0)	106 (35.6)	186 (33.2)	
Severe or extreme	21 (2.4)	9 (3.0)	12 (2.1)	
Digestive and endocrine				
None or mild	338 (39.4)	144 (48.3)	194 (34.6)	<0.001
Moderate	479 (55.8)	142 (47.7)	337 (60.2)	
Severe or extreme	41 (4.8)	12 (4.0)	29 (5.2)	
Genitourinary and reproductive				
None or mild	259 (30.3)	92 (30.9)	167 (29.9)	0.463
Moderate	181 (21.1)	56 (18.8)	125 (22.4)	
Severe or extreme	416 (48.6)	150 (50.3)	266 (47.7)	
Neuromusculoskeletal and movement				
None or mild	259 (30.2)	108 (36.2)	151 (27.0)	0.009
Moderate	511 (59.6)	157 (52.7)	354 (63.2)	
Severe or extreme	88 (10.3)	33 (11.1)	55 (9.8)	
Skin				
None or mild	695 (82.3)	244 (83.3)	451 (81.9)	0.875
Moderate	88 (10.4)	29 (9.9)	59 (10.7)	
Severe or extreme	61 (7.2)	20 (6.8)	41 (7.4)	

*P-value for heterogeneity

### Physical activity and chronic health conditions

PA, as measured by energy expenditure, varied among participants with different chronic health conditions (Table 3). For example, looking at adjusted results, those with dementia had an approximately 55% (*aMR* 0.45, 95% Confidence Interval CI 0.31–0.66) lower weekly energy expenditure than did participants without dementia, while heart patients had a 46% (*aMR* 0.54, CI 0.34–0.87) lower energy expenditure than did their counterparts. In contrast, participants with arthritis/osteoarthritis expended 27% (*aMR* 1.27, CI 1.07–1.51) more energy than did those without this condition. These results did not substantially differ when adjusted for cognitive level (results not shown).

**Table 3:** Chronic health conditions and physical activity energy expenditure (Kcal/week)

Chronic health condition (%)	Prevalence (%)	*cMR* ^a^	*aMR* ^b^ (95% CI)
Arthritis/Osteoarthritis	49.3	1.06	1.27 (1.07–1.51)
Hypertension	46.1	0.95	1.16 (0.97–1.39)
Diabetes	16.6	0.99	1.08 (0.86–1.36)
Depression	18.1	1.01	1.05 (0.84–1.31)
Arrhythmia	12.5	0.77	0.95 (0.71–1.26)
Thyroid disease	8.8	1.01	1.01 (0.75–1.36)
COPD	7.9	0.80	0.84 (0.61–1.16)
Ischemic heart disease	8.7	0.67	0.82 (0.61–1.10)
Anxiety	8.6	1.26	1.14 (0.84–1.55)
Cerebrovascular disease	11.3	0.62	0.95 (0.71–1.28)
Urinary incontinence	5.9	0.68	0.96 (0.66–1.40)
Cancer	5.8	1.31	1.14 (0.79–1.63)
Dementia	7.4	0.68	0.45 (0.31–0.66)
Neurodegenerative diseases	2.9	0.77	1.43 (0.83–2.48)
Visual alterations	7.3	0.45	0.85 (0.59–1.24)
Renal insufficiency	3.6	0.52	0.82 (0.52–1.32)
Heart failure	3.5	0.49	0.54 (0.34–0.87)
Peripheral artery disease	1.7	0.60	0.76 (0.42–1.39)
Anemia	4.4	0.95	1.09 (0.71–1.67)
Chronic hepatic disease	0.8	1.07	1.11 (0.45–2.73)
Severe mental disease	1.7	0.53	0.89 (0.43–1.84)
Deafness	4.2	0.45	0.76 (0.49–1.19)
Hip fracture	2.4	0.48	1.11 (0.59–2.09)
Asthma	3.1	0.76	0.89 (0.55–1.42)

*cMR* crude mean ratio, *CI* confidence interval; *aMR* adjusted mean ratio, *COPD* Chronic Obstructive Pulmonary Disease. The reference group is made up of persons free of the specific health condition

^a^Ratio of mean physical activity expenditure (Kcal/week) on comparing subjects with each chronic condition to those not suffering from the condition, obtained from negative binomial models

^b^Ratio of mean physical activity energy expenditure (Kcal/week) on comparing subjects with each chronic condition to those not suffering from the condition, obtained from negative binomial models, adjusted for sex, age (years), urban or rural setting, institutionalization (yes/no), number of chronic diseases (other than index condition), and cognitive status (MEC score, except in the case of dementia; MEC is the Spanish version of the Mini-Mental Status Examination)

### Physical activity, ICF body functions and WHODAS 2.0 disability

Looking at the adjusted estimates of the association between ICF body functions or WHODAS 2.0 disability and PA in terms of energy expenditure (Table 4), subjects with severe/extreme impairment in mental functions had a 62% lower weekly energy expenditure than did those with no/mild impairment, with a similar trend being in evidence for neuromusculoskeletal-and-movement functions (50% less expenditure). In the same direction, albeit less marked, the identical relationship was also observed for genitourinary-and-reproductive function, with 18% less expenditure *(aMR* 0.82). Finally, with regard to the association with PA, people with severe to extreme levels of WHODAS 2.0 disability (*aMR* 0.55) ranked close to those shown above for mental function impairment.

**Table 4:** ICF Checklist body functions, WHODAS 2.0 disability, and physical activity energy expenditure (Kcal/week)

ICF Body Functions and WHODAS disability level	*cMR* ^a^	*aMR* ^b^ (95% CI)
Mental		
Moderate	0.74	0.89 (0.73–1.10)
Severe or extreme	0.14	0.38 (0.21–0.68)
Sensory and pain		
Moderate	1.04	1.24 (0.95–1.63)
Severe or extreme	0.69	1.13 (0.81–1.59)
Voice and speech		
Moderate	0.74	1.16 (0.77–1.76)
Severe or extreme	0.52	1.32 (0.84–2.08)
Cardiovascular and respiratory		
Moderate	0.86	1.03 (0.84–1.25)
Severe or extreme	0.82	1.28 (0.72–2.28)
Digestive and endocrine		
Moderate	0.97	1.02 (0.85–1.22)
Severe or extreme	0.97	1.10 (0.72–1.69)
Genitourinary and reproductive		
Moderate	0.88	0.88 (0.70–1.11)
Severe or extreme	0.70	0.82 (0.67–1.00)
Neuromusculoskeletal and movement		
Moderate	0.93	0.99 (0.81–1.21)
Severe or extreme	0.28	0.50 (0.35–0.72)
Skin		
Moderate	0.94	0.89 (0.68–1.16)
Severe or extreme	0.63	0.84 (0.58–1.20)
WHODAS 2.0 disability		
Moderate	0.71	0.80 (0.50–1.27)
Severe to complete	0.36	0.55 (0.34–0.91)

*cMR* crude mean ratio, *CI* confidence interval, *aMR* adjusted mean ratio, *WHODAS* World Health Organization Disability Assessment Schedule. The reference groups are “none or mild impairment” for body functions, and “no or mild disability” for WHODAS 2.0

^a^Ratio of mean physical activity expenditure (Kcal/week), on comparing subjects within each level of impairment to those of the reference category (no/mild impairment), obtained from negative binomial models

^b^Ratio of mean physical activity energy expenditure (Kcal/week), on comparing subjects within each level of impairment to those of the reference category (no/mild impairment), obtained from negative binomial models, adjusted for sex, age (years), urban or rural setting, institutionalization (yes/no), number of chronic diseases, cognitive status (MEC score, the Spanish version of the Mini-Mental Status Examination) and degree of impairment in the remaining body functions (in continuous form, with values from 0 to 100%). We followed a similar approach with disability, as measured by the WHODAS 2.0 score

The sensitivity analysis performed with total time of physical activities yielded largely similar results (Table 5). In the analysis restricted to a sample of individuals without dementia, the results shown in Tables 4 and 5 proved quite similar.

**Table 5:** Impairment in ICF Checklist body functions, WHODAS 2.0 disability and physical activity (hours/week)

ICF Body Functions and WHODAS disability level	*cMR* ^a^	*aMR* ^b^ (95% CI)
Mental		
Moderate	0.72	0.90 (0.79–1.04)
Severe or extreme	0.14	0.37 (0.23–0.57)
Sensory and pain		
Moderate	1.04	1.28 (1.07–1.55)
Severe or extreme	0.71	1.20 (0.95–1.51)
Voice and speech		
Moderate	0.68	1.00 (0.74–1.34)
Severe or extreme	0.51	1.09 (0.80–1.50)
Cardiovascular and respiratory		
Moderate	0.87	1.00 (0.87–1.14)
Severe or extreme	0.76	1.09 (0.73–1.62)
Digestive and endocrine		
Moderate	0.94	0.95 (0.84–1.09)
Severe or extreme	0.78	0.81 (0.60–1.09)
Genitourinary and reproductive		
Moderate	0.93	0.94 (0.81–1.11)
Severe or extreme	0.69	0.86 (0.75–0.99)
Neuromusculoskeletal and movement		
Moderate	0.91	0.99 (0.86–1.13)
Severe or extreme	0.24	0.54 (0.42–0.71)
Skin		
Moderate	0.93	0.93 (0.77–1.13)
Severe or extreme	0.62	0.96 (0.74–1.25)
WHODAS 2.0 disability		
Moderate	0.79	0.89 (0.66–1.20)
Severe to complete	0.40	0.61 (0.44–0.85)

*cMR* crude mean ratio, *CI* confidence interval, *aMR* adjusted mean ratio, *WHODAS* World Health Organization Disability Assessment Schedule. The reference groups are “none or mild impairment” for body functions, and “no or mild disability” for WHODAS 2.0

^a^Ratio of mean number of hours per week of subjects within each level of impairment to those of the reference category (no/mild impairment), obtained from negative binomial models

^b^Ratio of mean number of hours per week of subjects within each level of impairment to those of the reference category (no/mild impairment), obtained from negative binomial models, adjusted for sex, age (years), urban or rural setting, institutionalization (yes/no), body mass index, number of chronic diseases, cognitive status (MEC score, the Spanish version of the Mini-Mental Status Examination), and degree of impairment in the remaining body functions (in continuous form, with values from 0 to 100%). We followed a similar approach with disability, as measured by the WHODAS 2.0 score

## Discussion

The association between specific disability components and PA in older adults was assessed using the ICF framework. In this study, PA was a composite of items selected from both activity and participation (measured in terms of time or energy expenditure, rather than difficulty of performance as contemplated by the ICF), and was therefore deemed to be a personal factor. Women reported engaging in PA for a longer time and expending more energy than did men. PA was negatively and significantly associated with mental, neuromusculoskeletal and movement function impairment, as well as with WHODAS 2.0 disability. In a different study on the same sample [15], disability was strongly determined by mental and neuromusculoskeletal function impairment. In addition, dementia and health failure determined low energy expenditure during PA, even after adjusting for confounders such as age and urban setting.

### Body functions, disability and physical activity

The association between mental function and PA has been widely studied. Although debated, a two-way causal relationship was proposed to explain the positive relationship between good mental health and PA [24]. Anhedonia may contribute to the effect of mental function impairment on PA [25] but other symptoms may also be involved. The findings suggest that, overall, multiple medical conditions may lead to mental function impairment and low PA. Associations may have been affected by recall bias, which would have led to an underestimation of PA in populations with mental disorders, including dementia. However, little physical activity among persons with mental health problems is consistent with the well-known high frequency of chronic conditions in this group [26] and with the high prevalence of patients with dementia within the group. Excluding patients diagnosed with dementia from the analysis did not substantially modify results.

Low weekly energy expenditure with severe or extreme neuromusculoskeletal- and movement-related function impairment in some way contradicts the high level of PA among persons with osteoarthritis. Moreover, PA is expected to improve neuromusculoskeletal- and movement-related functions [27, 28]. To speculate, and despite the fact that our cross-sectional design does not formally allow for causal interpretations, a low prevalence of dementia among rheumatoid arthritis patients treated with anti-inflammatory drugs might make for a moderately higher-than-expected level of PA [29].

Genitourinary and reproductive function impairment was modestly associated with a lower level of PA. This finding is consistent with epidemiologic studies which describe a lower frequency of genitourinary cancer (prostate, bladder, renal cell, and testicular) in physically more active persons [30]. In contrast, a meta-analysis of studies showed an improvement in prostatic hyperplasia or lower urinary tract symptoms among men with increased PA [31].

In our study, associations with WHODAS 2.0 were weaker than those with mental or neuromusculoskeletal and movement body functions, suggesting that a proportion of the risk of low PA associated with mental or neuromusculoskeletal and movement function impairment might be channeled through performance in activity and participation. Indeed, work, mobility, and care taking are dimensions of the YPAS, and they are tasks or actions that overlap with activities and participation in the ICF. The reportedly low self-care performance of heart-failure patients potentially not complying with therapies may serve as a good example of the complex relationships between health conditions, loss of functions, disability and PA [32]. Further studies are needed to analyze the role of contextual factors, such as physical and social barriers, in reducing PA in persons with mental and non-inflammatory musculoskeletal disorders. Our crude or adjusted mean ratios might possibly be underestimated, since the reference category included persons with mild impairment or disability, as well as persons underdiagnosed with dementia.

### Implications of methodology in outcome measurements

Energy expenditure during PA was our primary outcome to measure PA, as recommended by Lamonte and Ainsworth, who identified various drawbacks of PA assessment via questionnaires [33]. They emphasized the need for an energy-expenditure metric to enable official recommendations on PA levels and between-studies comparison. The YPAS yields results on both PA duration and energy expenditure. To allow for cross-cultural specificity, we used a validated Spanish version of the YPAS, which reliably measures PA in older adults [18]. In the 108 independent Spanish community-dwelling elderly subjects (ages 61 through 80 years) included in the validation study, YPAS energy expenditure was 10,598 kcal/week among men and 12,237 kcal/week among women [18]. There are two main reasons to account for this low energy expenditure: 1) people screened negative for disability were excluded from the sample; and, 2) 55.0% of those who screened positive were moderately and severely/extremely disabled. The YPAS gender difference could be explained by a higher intensity during PA and overrepresentation of activities with high energy expenditure among men, even though PA duration was lower.

The YPAS uses an absolute measurement of PA intensity, since a relative measure (e.g., light, vigorous, etc.) would be age-dependent: a similar dose of PA represents a different intensity for a 55-year-old or a 90-year-old person. Intensity was taken into account in our analysis when adjusting for age. We likewise adjusted for gender. Since self-reported instruments fail to show sufficient reliability or validity [34], our questionnaire was administrated by trained investigators. As regards the possibility of recall bias, especially in the case of older adults with cognitive impairment, it should be noted that the time interval was short (latest week’s PA), and that cognitive impairment was taken into account in the multivariate analysis.

Results from our study call for further research in several areas. Firstly, this paper reports on data gathered from 2008 through 2011, coinciding with the Spanish economic crisis, a phenomenon that might conceivably have had a negative impact on health outcomes [35]. Continuing work is needed as new data become available. As the ICF qualifiers are not operationalized in clear, observational indicators, the reliability of the scoring system needs to be addressed by a purpose-designed study. Values were however assigned by trained researchers in our study [2]. Since disability, a strong predictor of low PA, also predicts the worsening of disability and death, a proportion of the protective effect of PA in longitudinal, observational studies may correspond to the effects of uncontrolled disability and, by extension, to bias overestimating the benefits of PA [36]. The cross-sectional nature of this study’s design means that causal inferences cannot be made: more research with longitudinal data is thus needed.

Our study also displays several strengths. The quality of measurement was high thanks to the use of trained field investigators and Spanish-validated, reliable, detailed instruments, such as the YPAS and WHODAS. Furthermore, the use of the ICF framework enables easy transposition to the clinical and functional field.

### Implications for preventive medicine and public health

Since the PA of older adults is a major concern, our findings may be useful for public health authorities, clinicians and rehabilitation specialists, in order to maintain or target specific body functions in the healthcare process. Prescription of individualized physical activity explicitly requires the absence of physical limitations to engaging in such activity, and should therefore be tailored to the individual’s function loss or disability, i.e., mobility [37]. Selected groups, i.e., excluding diagnostic categories or addressing groups with low PA but preserved mobility and mental functions, might constitute preferential targets for intervention aimed at reducing a sedentary life style in primary care programs. Technical support and correction of incontinence may also be indicated, particularly if patients aim to improve PA. In the ICF framework, the level of performance is estimated by taking into account any environmental factors that may modulate achievements, i.e., by eliminating barriers (such as stairs for altered mobility) or introducing facilitators (a wheelchair, for example, or appropriate community programs for complementary physical activity). Further studies including evaluation of environment-based interventions could help assess strategies’ effectiveness. Lastly, our results may stimulate PA research covering the existing gap between preventive medicine, which is individually-oriented by definition, and public health. In the latter case, pending tasks include the identification and characterization of potential high-risk groups among older adults (e.g., non-institutionalized, non-homebound, mild or moderately disabled, with low energy expenditure) and the design of tailored, evidence-based recommendations for such groups.

## Conclusions

Mental as well as neuromusculoskeletal impairments and disability were the strongest factors associated with low PA among older adults. These body functions and related health conditions, such as dementia and heart failure, may constitute a specific target for public health and clinical interventions aimed at improving the PA of middle-aged and older adults. Links between disability and PA may have theoretical implications for research methods in a life-course context.

## Additional file

Additional file 1: Part 1a of the ICF Checklist used in the study (text and table). (DOCX 16 kb)

## Abbreviations

aMR: adjusted Mean Ratio
BMI: Body Mass Index
CI: Confidence Interval
cMR: crude Mean Ratio
COPD: Chronic Obstructive Pulmonary Disease
ICF: International Classification of Functioning, Disability and Health
Kcal: Kilocalories
M: Mean
MEC: Mini Examen Cognoscitivo (Spanish version of the Mini-Mental Status Examination)
MMSE: Mini-Mental Status Examination
PA: Physical Activity
SD: Standard Deviation
WHODAS 2.0: World Health Organization Disability Assessment Schedule
YPAS: Yale Physical Activity Survey
