# Supplementary material for: Physical activity, body functions and disability among middle-aged and older Spanish adults
Source: BMC Geriatr. 2017 Jul 18;17:150. doi: 10.1186/s12877-017-0551-z (PMC5516319; doi:10.1186/s12877-017-0551-z)
Supplement: Additional file 1: — Part 1a of the ICF Checklist used in the study (text and table). (DOCX 16 kb) [file 12877_2017_551_MOESM1_ESM.docx]

**Additional file 1**

**Part 1a of the ICF Checklist used in the study**

The International Classification of Functioning, Disability and Health (ICF) and the ICF Checklist are available at: <http://www.who.int/classifications/icf/icfchecklist.pdf?ua=1> (latest access, 8 September 2015).

**PART 1a: IMPAIRMENTS of BODY FUNCTIONS**

Body functions are the physiological functions of body systems (including psychological functions).

Impairments are problems in body function as a significant deviation or loss*.*

***First Qualifier:*** *Extent of impairments*

***0 No impairment*** means the person has no problem

***1 Mild impairment*** means a problem that is present less than 25% of the time, with an intensity a person can tolerate and which happens rarely over the last 30 days.

***2 Moderate impairment*** means that a problem that is present less than 50% of the time, with an intensity, which is interfering in the persons day to day life and which happens occasionally over the last 30 days.

***3 Severe impairment*** means that a problem that is present more than 50% of the time, with an intensity, which is partially disrupting the persons day to day life and which happens frequently over the last 30 days.

***4 Complete impairment*** means that a problem that is present more than 95% of the time, with an intensity, which is totally disrupting the persons day to day life and which happens every day over the last 30 days.

***8 Not specified*** means there is insufficient information to specify the severity of the impairment.

***9 Not applicable*** means it is inappropriate to apply a particular code (e.g. b650 Menstruation functions for woman in pre-menarche or post-menopause age).

| ***Short List of Body Functions*** | ***Qualifier*** |
| --- | --- |
| **b1. MENTAL FUNCTIONS** |  |
| **b110** Consciousness |  |
| **b114** Orientation *(time, place, person)* |  |
| **b117** Intellectual *( incl. Retardation, dementia)* |  |
| **b130** Energy and drive functions |  |
| **b134** Sleep |  |
| **b140** Attention |  |
| **b144** Memory |  |
| **b152** Emotional functions |  |
| **b156** Perceptual functions |  |
| **b164** Higher level cognitive functions |  |
| **b167** Language |  |
| **b2. SENSORY FUNCTIONS AND PAIN** |  |
| **b210** Seeing |  |
| **b230** Hearing |  |
| **b235** Vestibular *(incl. Balance functions)* |  |
| **b280** Pain |  |
| **b3. VOICE AND SPEECH FUNCTIONS** |  |
| **b310** Voice |  |
| **b4. FUNCTIONS OF THE CARDIOVASCULAR, HAEMATOLOGICAL, IMMUNOLOGICAL AND RESPIRATORY SYSTEMS** |  |
| **b410** Heart |  |
| **b420** Blood pressure |  |
| **b430** Haematological *(blood)* |  |
| **b435** Immunological *(allergies, hypersensitivity)* |  |
| **b440** Respiration *(breathing)* |  |
| **b5. FUNCTIONS OF THE DIGESTIVE, METABOLIC AND ENDOCRINE SYSTEMS** |  |
| **b515** Digestive |  |
| **b525** Defecation |  |
| **b530** Weight maintenance |  |
| **b555** Endocrine glands *(hormonal changes)* |  |
| **b6. GENITOURINARY AND REPRODUCTIVE FUNCTIONS** |  |
| **b620** Urination functions |  |
| **b640** Sexual functions |  |
| **b7. NEUROMUSCULOSKELETAL AND MOVEMENT RELATED FUNCTIONS** |  |
| **b710** Mobility of joint |  |
| **b730** Muscle power |  |
| **b735** Muscle tone |  |
| **b765** Involuntary movements |  |
| **b8. FUNCTIONS OF THE SKIN AND RELATED STRUCTURES** |  |
| **ANY OTHER BODY FUNCTIONS** |  |
